# Association of diabetes with severity and mortality in hospitalized patients with COVID-19 in Wuhan, China: a single-centered, retrospective study

**DOI:** 10.20945/2359-3997000000384

**Published:** 2021-07-16

**Authors:** You-ping Deng, Wen Xie, Tao Liu, Shou-yi Wang, Yu-xing Zan, Mei-rong Wang, Xiao-bo Meng, Jie Zheng, Hai-rong Xiong, Xue-dong Fu

**Affiliations:** 1 Wuhan University Zhongnan Hospital Department of Pediatrics Wuhan P.R. China Department of Pediatrics, Zhongnan Hospital of Wuhan University, Wuhan, P.R. China; 2 Wuhan University Zhongnan Hospital Department of Clinical Laboratory Wuhan P.R. China Department of Clinical Laboratory, Zhongnan Hospital of Wuhan University, Wuhan, P.R. China.; 3 Wuhan University Zhongnan Hospital Department of Urology Wuhan P.R. China Department of Urology, Zhongnan Hospital of Wuhan University, Wuhan, P.R. China.; 4 Wuhan University School of Basic Medical Sciences Institute of Medical Virology Wuhan P.R. China State Key Laboratory of Virology/Institute of Medical Virology, School of Basic Medical Sciences, Wuhan University, Wuhan, P.R. China.

**Keywords:** COVID-19, diabetes, mortality, clinical characteristics

## Abstract

**Objective:**

Coronavirus disease 2019 (COVID-19), caused by severe acute respiratory syndrome coronavirus 2 (SARS-CoV-2), has spread worldwide. The aim this study was to investigate the association of diabetes with severity and mortality among hospitalized patients with COVID-19 in Wuhan, China.

**Subjects and methods:**

This retrospective, single-center case study enrolled a total of 564 patients diagnosed with COVID-19 at the Seventh Hospital of Wuhan City, between January 20 and March 15, 2020.

**Results:**

Among the 564 patients with confirmed COVID-19, 509 (85.1%) were discharged and 55 (9.8%) died. The median age was 59 years (range, 10-93 years). A total of 85 (15.1%) patients were diagnosed with diabetes on admission (median age, 65.0 [range, 34-91] years). Patients with diabetes had significantly higher proportions of critical cases (24 [28.2%] vs. 66 [13.8%]) and in-hospital mortality (17 [20%] vs. 38 [7.9%]). Moreover, patients with diabetes presented abnormal levels of multiple indicators concerning lymphopenia, inflammation, heart, liver, kidney, and lung function on admission, while diabetic patient group still display higher troponin T (TnT) levels when approaching discharge. The Kaplan-Meier survival curve indicated a trend toward poorer survival in diabetic patients compared to non-diabetic patients, also evidenced by abnormal laboratory biomarker changes regarding multiple system impairments among COVID-19 patients with diabetes with in-hospital death.

**Conclusion:**

The detailed clinical investigation of 564 hospitalized patients with COVID-19 indicated a considerable association between diabetes and COVID-19 severity or mortality. Thus, more intensive treatment may be considered for COVID-19 patients with diabetes, especially regarding to cardiac injury.

## INTRODUCTION

Novel coronavirus disease 2019 (COVID-19) is caused by the severe acute respiratory syndrome coronavirus 2 (SARS-CoV2), which is a newly emerged envelope RNA β-coronavirus with a round/oval shape and a diameter of 60-140 nm (1,2). The COVID-19 outbreak has spread worldwide, endangering global public health (3,4). SARS-CoV2 infection can induce clinical symptoms including fever, dry cough, dyspnea, and fatigue, and ultimately result in acute respiratory distress syndrome (ARDS), septic shock, and multiple organ dysfunction syndrome with high morbidity and mortality (1). Early reports suggested that patients with certain comorbid conditions, such as hypertension, cardiovascular disease, and diabetes, may face higher risks and are more likely to develop severe COVID-19 (3,5).

Diabetes mellitus is a complex chronic illness that is associated with considerable morbidity and mortality worldwide (6). Patients with diabetes are more sensitive to infections and may have a poor prognosis compared to that in patients without diabetes, which may be due to the impairment of their immune status (7). Diabetes has been reported to be one of the most frequent comorbidities among patients with COVID-19 (8,9). Therefore, we retrospectively reviewed the clinical data from a single center in Wuhan, China, and assessed the association between diabetes and COVID-19. We compared the differences in clinical characteristics, laboratory findings, treatment, and outcomes between diabetic patients and non-diabetic patients, as well as those indicators among the survivors and non-survivors in patients with diabetes, which may provide a hint for the clinical management of diabetic patients with COVID-19.

## SUBJECTS AND METHODS

### Study design

This study was conducted at the No.7 Hospital of Wuhan, a government-designated that was consigned to Zhongnan Hospital of Wuhan University during the COVID-19 pandemic. A total of 564 patients with confirmed COVID-19 hospitalized at the No.7 Hospital of Wuhan were enrolled in this study, which was conducted from January 20 to March 15, 2020. All patients were diagnosed with COVID-19 and classified into distinct clinical types according to the diagnostic and treatment guidelines of COVID-19 from the Chinese National Health Commission (version 3-7) (10). According to the National Public Health Emergency Management System, patients with mild-type COVID-19 were treated at Fangcang shelter hospitals (11,12). Thus, all patients involved in this study were in moderate, severe, or critical condition. Patients with diabetes were verified through medical records or self-reported diagnoses reviewed by their physicians. This study was approved by the institutional ethics board of Zhongnan Hospital, Wuhan University (No.2020056K), which waived the requirement for written informed consent for the emerging infectious disease.

### Data collection

The medical records, including basic information (age, sex, comorbidities, etc.), clinical characteristics, laboratory findings, radiological examinations, treatment, and outcomes of each patient were obtained from their medical records. The date of disease onset was defined as the day on which symptoms were noticed. Pharyngeal swab specimens were collected for laboratory viral nucleic acid detection of SARS-CoV-2 using quantitative reverse-transcription-polymerase chain reaction (qRT-PCR) (13). The patient samples were also tested for other viral pathogens, including influenza virus, parainfluenza, Coxsackie virus, adenovirus, echovirus, respiratory syncytial virus, and cytomegalovirus. All patients underwent chest computed tomography (CT) or X-ray radiography. Follow-up radiological examination and negative SARS-CoV-2 test results were considered reference indices for cure and hospital discharge.

Laboratory examinations conducted at admission and with disease progression included routine blood tests and assessments of blood biochemistry, blood gas level, blood electrolytes, coagulation function, procalcitonin (PCT), C-reactive protein (CRP), serum amyloid A (SAA), serum creatine kinase and myocardial enzyme spectrum. Medical treatments were recorded, including antiviral treatment, Chinese patent medicine, corticosteroids, gamma globulin, probiotics, etc. Treatment strategies, such as supplemental oxygen, noninvasive mechanical ventilation, or invasive mechanical ventilation, were also recorded.

### Statistical analysis

Non-normally distributed continuous data were described using median and interquartile range (IQR) values, while categorical data were expressed as numbers/frequencies and percentages. Chi-square and Fisher's exact tests were used to compare the frequencies of the categorical variables. Continuous variables were tested for Gaussian distribution by D’Agostino-Pearson omnibus normality test and further analyzed using Mann-Whitney tests as appropriate. The association between diabetes and death was quantified by logic regression after adjusting for controlled confounders. The E-values were then calculated to assess the robustness of the associations to potential unmeasured confounders using “EValue” package in software R (14). Survival curves were generated using the Kaplan-Meier method, and comparisons between groups were performed using log-rank tests. Landmark analyses were performed using EmpowerStats (http://www.empowerstats.com) and the statistical package R. Other statistical analyses were performed using GraphPad Prism version 6.00 software (GraphPad Software Inc.). *P* value less than .05 was considered statistically significant.

## RESULTS

### Demographics and clinical characteristics

**Figure 1:**
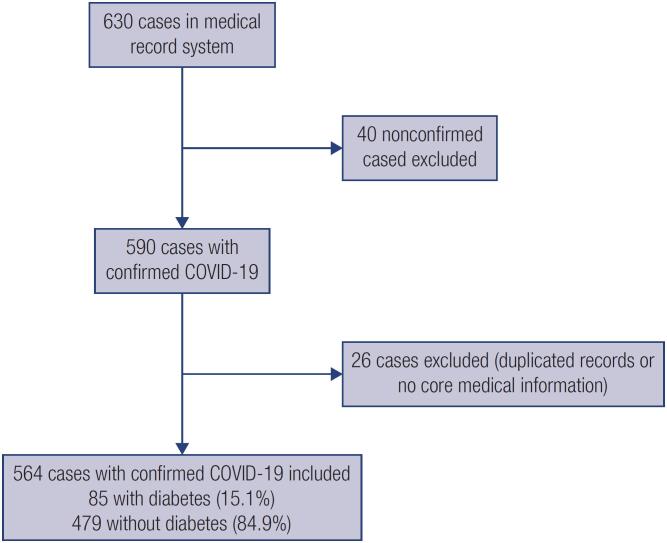
Flowchart of patient recruitment.

The flowchart in Figure 1 shows the patient selection in this study. Briefly, a total 630 of patients in the medical record system were screened from January 20 to March 15, 2020, among which 40 patients were not confirmed and 26 patients without core medical information and duplicated records were excluded. Thus, the present study included a total of 564 patients hospitalized with confirmed COVID-19, including 85 (15.1%) patients diagnosed with diabetes on admission. The median age of all patients was 59 years (range,10-93 years), and 275 (50.7%) patients were male. The most common underlying comorbidities were hypertension (n = 190, 33.7%), cardiovascular disease (n = 70, 12.4%), and liver disease (n = 33, 5.9%). Among the 564 patients, 284 (50.4%), 190 (33.7%), and 90 (16.0%) were categorized as having moderate, severe, and critical statuses. Of these 564 patients, 509 (90.2%) were discharged and 55 (9.8%) died.

Compared to patients without diabetes, patients with diabetes were older (median [range] age, 65 [34-91] vs. 57 [10-93] years; *p* < 0.0001) and most were male (49, 57.6%). Moreover, patients with diabetes presented significantly higher rates of comorbidities, including hypertension (58 [68.2%] vs. 132 [27.6%], *p* < 0.0001) and cardiovascular disease (25 [29.4%] vs. 45 [9.4%], *p* < 0.0001). Patients with diabetes had a significantly higher proportion of critical cases (24 [28.2%] vs. 66 [13.8%], *p* = 0.0019). Mortality was also significantly higher in patients with diabetes (17 [20.0%] vs. 38 [7.9%]) (Table 1).

**Table 1:** Demographics and clinical characteristics of patients with COVID-19

Characteristic		No. (%)			
		Total (n = 564)	Non-diabetes (n = 479)	Diabetes (n = 85)	*P* value
Age-median(range)		59 (10-93)	57 (10-93)	65 (34-91)	<0.0001
Sex					
	Female	289 (51.2)	253 (52.8)	36 (42.4)	0.0786
	Male	275 (48.8)	226 (47.2)	49 (57.6)	
Smoking		42 (7.4)	38	4	0.3745
Onset of symptom to hospital admission, median (IQR), d		10 (6-15)	10 (6-15)	10 (7-15)	0.7271
Hospitalization, median (IQR), d		15 (9-24)	15 (9-23)	17 (10-25)	0.3302
Comorbidity – No. (%)					
	Cardiovascular disease	70 (12.4)	45 (9.4)	25 (29.4)	<0.0001
	Cerebrovascular disease	11 (2.0)	7 (1.5)	4 (4.7)	0.0685
	Hypertension	190 (33.7)	132 (27.6)	58 (68.2)	<0.0001
	Chronic bronchitis	12 (2.1)	11 (2.3)	1 (1.2)	1
	Malignancy	24 (4.3)	18 (3.8)	6 (7.1)	0.2364
	Liver disease	33 (5.9)	29 (6.1)	4 (4.7)	0.8038
	Kidney disease	25 (4.4)	19 (4.0)	6 (7.1)	0.2461
	Allergic physique	19 (3.4)	15 (3.1)	4 (4.7)	0.5092
Complication					
	Bacterial infection	42 (7.4)	35 (7.3)	7 (8.2)	0.8220
	Metabolic acidosis	19 (3.4)	11 (2.3)	8 (9.4)	0.0036
	Heart failure	25 (4.4)	15 (3.1)	10 (11.8)	0.0017
	ARDS	61 (10.8)	41 (8.6)	20 (23.5)	0.0002
	Acute liver injury	24 (4.3)	18 (3.8)	6 (7.1)	0.2364
	Acute kidney injury	27 (4.8)	19 (4.0)	8 (9.4)	0.0477
	DIC	7 (1.2)	3 (0.6)	4 (4.7)	0.0118
Treatments					
	Antiviral treatment	351 (62.2)	299 (62.4)	52 (61.2)	0.9034
	Antibiotics	407(72.1)	341 (71.1)	66 (77.6)	0.2397
	Chinese Medicine	343 (60.8)	289 (60.3)	54 (63.5)	0.6305
	Glucocorticoid	193 (34.2)	159 (33.2)	34 (40.0)	0.2641
	Immune globulin	90 (16.0)	70 (14.6)	20 (23.5)	0.0524
Respiratory support					0.0462
	Nasal cannula	305 (54.1)	257 (53.7)	48 (56.5)	
	Non-invasive ventilation	35 (6.2)	25 (5.2)	10 (11.8)	
	Invasive ventilation	15 (2.7)	10 (2.1)	5 (5.9)	
Diseases severity					0.0018
	Moderate	284 (50.4)	252 (52.6)	32 (37.6)	
	Severe	190 (33.7)	161 (33.6)	29 (34.1)	
	Critical	90 (16.0)	66 (13.8)	24 (28.2)	
Clinical outcomes					0.0022
	Discharge	509 (90.2)	441 (92.1)	68 (80.0)	
	Death	55 (9.8)	38 (7.9)	17 (20.0)	

ARDS: acute respiratory distress syndrome; DIC: disseminated intravascular coagulation; IQR: interquartile range.

### Laboratory findings on admission

As shown in Table 2, in the overall study population of 564 patients, the median levels of C-reactive protein (CRP) (20.30 [2.50-59.59] mg/L), and serum amyloid A (74.9 [12.0-173.1] mg/L) were elevated, while those of lymphocyte count (1.02 [0.65-1.50] x10^9^/L), total protein (64.1 [60.3-67.8] g/L), and albumin (37.4 [33.1-40.8] g/L) were decreased. However, the other laboratory indicators were within the normal ranges, including other blood cell counts, blood lipids and electrolytes, cardiac biomarkers, blood gas analysis, and other biomarkers of liver and renal function.

**Table 2:** Laboratory results among different groups

Characteristic		Median (IQR)			
		Total (n = 564)	Non-diabetes (n = 479)	Diabetes (n = 85)	*P* value
Blood cell count					
	White blood cell count, ×10^9^/L (normal range 3.5-9.5)	5.02 (3.94-6.93)	4.97 (3.90-6.81)	5.95 (4.36-7.47)	0.0781
	Neutrophil count, ×10^9^/L (normal range 1.6-6.3)	3.30 (2.38-5.01)	3.17 (2.31-4.80)	3.88 (3.00-6.01)	0.0020
	Lymphocyte count, ×10^9^/L (normal range 1.1-3.2)	1.02 (0.65-1.50)	1.05 (0.67-1.53)	0.82 (0.60-1.34)	0.0153
	Monocyte count, ×10^9^/L (normal range 0.1-0.6)	0.39 (0.29-0.51)	0.39 (0.29-0.51)	0.36 (0.25-0.52)	0.1179
	Platelet count, ×10^9^/L (normal range 125-350)	190.0 (141.5-244.5)	193.0 (142.0-246.5)	181.0 (128.0-233.5)	0.1463
Blood lipids and electrolytes					
	Total Cholesterol, mmol/L (normal range 2.8-5.2)	3.74 (3.12-4.36)	3.89 (3.00-4.32)	3.38 (2.87-4.35)	0.0206
	Triglyceride, mmol/L (normal range 0.56-1.7)	0.95 (0.71-1.39)	0.93 (0.70-1.38)	1.05 (0.76-1.69)	0.1098
	HDL, mmol/L (normal range 0.9-2.1)	1.13(0.95-1.36)	1.14 (0.97-1.32)	1.08 (0.84-1.24)	0.0055
	LDL, mmol/L (normal range 1-3.35)	2.12(1.70-2.63)	2.15 (1.73-2.65)	1.92 (1.57-2.56)	0.0364
	sdLDL, mmol/L (normal range 95-538)	137.5 (95.8-213.0)	138.0 (94.8-213.0)	134.5 (97.0-215.3)	0.9037
Serum					
	Potassium, mmol/L (normal range 3.5-5.3)	3.82 (3.46-4.18)	3.80 (3.46-4.15)	3.89 (3.53-4.45)	0.0997
	Calcium, mmol/L (normal range 2.11-2.52)	2.19 (2.09-2.31)	2.19 (2.10-2.31)	2.16 (2.00-2.29)	0.0589
Inflammatory biomarkers					
	hsCRP, mg/L (normal range 0-3)	20.30 (2.50-58.59)	18.0 (2.15-55.0)	34.6 (6.68-81.73)	0.0045
	Procalcitonin, ng/mL (normal range 0-0.1)	0.06 (0.04-0.17)	0.06 (0.04-0.16)	0.09 (0.05-0.26)	0.0941
	SAA, mg/L (normal range 0-10)	74.9 (12.0-173.1)	87.6 (12.0-206.5)	34.7 (10.2-90.92)	0.1308
Cardiac biomarkers					
	TnT, ng/mL (normal range 0-0.014)	0.009 (0.006-0.0140)	0.008 (0.006-0.013)	0.013 (0.009-0.032)	<0.0001
	Creatine kinase-MB, ng/mL (normal range 0-6.22)	1.16 (1.00-2.49)	1.19 (0.76-2.22)	1.73 (0.97-3.51)	0.0050
	Myoglobulin, ng/mL (normal range 7.4-105.7)	43.1 (27.2-78.4)	42.5(26.2-76.1)	49.6 (27.2-146.5)	0.0121
	NT-proBNP, pg/mL (normal range 0-222)	157.5 (50.2-438.5)	134.9 (48.6-422.9)	271.9 (99.8-618.0)	0.0037
Blood gas analysis					
	PaO_2_, mmHg (normal range 70-107)	87.0 (66.0-118.5)	92.0 (69.0-120.5)	74.5 (55.3-106.3)	0.0122
	PaO_2_/FiO_2_, mmHg	376.0 (232.0-481.0)	385.5 (248.0-491.3)	290.5 (163.6-400.0)	0.0014
	PaCO_2_, mmHg (normal range 35-45)	39.0 (34.0-45.0)	40.0 (34.0-45.0)	36.0 (31.5-45.5)	0.2094
	PH (normal range 7.35-7.45)	7.42 (7.39-7.45)	7.42 (7.39-7.45)	7.42 (7.38-7.45)	0.5954
	BE, mmol/L (normal range -3-3)	1.40 (-0.50-3.1)	1.50 (-0.10-3.10)	0.50 (-2.55-2.90)	0.0179
Liver and renal function					
	Alanine Aminotransferase, IU/L (normal range 9-50)	23.0 (15.0-36.0)	23.0 (15.0-35.9)	24.0 (15.0-38.5)	0.5668
	Aspartate aminotransferase, IU/L (normal range 15-40)	26.0 (18.0-37.0)	25.5 (18.0-37.0)	26.5 (17.8-38.3)	0.8051
	Total protein, g/L (normal range 65-85)	64.1 (60.3-67.8)	64.1 (60.5-67.7)	63.7 (59.3-68.5)	0.6828
	Albumin, g/L (normal range 40-55)	37.4 (33.1-40.8)	37.6 (33.5-41.0)	34.8 (30.9-39.3)	0.0055
	Globulin, g/L (normal range 20-40)	26.5 (23.9-30.0)	26.4 (23.7-29.7)	27.9 (25.8-32.5)	0.0031
	Total bilirubin, μmol/L (normal range 2-23)	7.9 (5.8-11.0)	7.9 (5.7-10.8)	9.2 (7.2-12.7)	0.0051
	Direct bilirubin, μmol/L (normal range 0-8)	3.0 (2.0-4.1)	3.0 (2.0-4.0)	3.8 (2.2-5.6)	0.0034
	Creatinine, μmol/L (normal range 57-97)	63.0 (53.0-74.3)	63.0 (53.0-73.0)	66.0 (54.0-79.0)	0.3139
	Urea nitrogen, μmol/L (normal range 3.1-8)	4.32 (3.40-5.81)	4.27 (3.36-5.56)	4.84 (3.63-6.47)	0.0183

HDL: high-density lipoprotein; sdLDL: small dense low-density lipoprotein; LDL: low-density lipoprotein; CRP: C-reactive protein; SAA: serum amyloid A; TnT: troponin T; CK-MB: creatine kinase-MB; NT-proBNP: N-terminal pro-brain natriuretic peptide.

Compared to patients without diabetes, patients with diabetes presented with significantly higher neutrophil counts (median [IQR], 3.88 [3.00-6.01] vs. 3.17 [2.31-4.80] x10^9^/L, *p* = 0.002), and lower lymphocyte counts (median [IQR], 0.82 [0.60-1.34] vs. 1.05 [0.67-1.53] x10^9^/L; *p* = 0.0153). The white blood cell, lymphocyte, and platelet counts of these two groups were similar.

Triglyceride and small dense low-density lipoprotein (sdLDL) levels did not differ between patients with and without diabetes, while patients with diabetes had lower levels of total cholesterol (median [IQR], 3.38 [2.87-4.35] vs. 3.89 [3.00-4.32] mmol/L; *p* = 0.0206), high-density lipoprotein (HDL) (median [IQR], 1.08 [0.84-1.24] vs. 1.14 [0.97-1.32] mmol/L; *p* = 0.0055), and low-density lipoprotein (LDL) (median [IQR], 1.92 [1.57-2.56] vs. 2.15 [1.73-2.65] mmol/L; *p* = 0.0364). Moreover, patients with diabetes had evidence of more severe respiratory dysfunction, with lower partial pressure of oxygen (PaO_2_) (median [IQR], 74.5 [55.3-106.3] vs. 92.0 [69.0-120.5] mmHg; *p* = 0.0122), and PaO_2_/fraction of inspired oxygen (FiO_2_) (median [IQR], 290.5 [163.3-400.0] vs. 385.5 [248.0-491.3] mmHg; *p* = 0.0014). Furthermore, patients with diabetes also had higher levels of urea nitrogen (median [IQR], 4.84 [3.63-6.47] vs. 4.27 [3.36-5.56] μmol/L; *p* = 0.0183). Patients with diabetes also presented higher level of total bilirubin (median [IQR], 9.2 [7.2-12.7] vs. 7.9 [5.7-10.8] μmol/L; *p* = 0.0051), direct bilirubin (median [IQR], 3.8 [2.2-5.6] vs. 3.0 [2.0-4.0] μmol/L; *p* = 0.0034), and lower albumin levels (median [IQR], 34.8 [30.9-39.3] vs. 37.6 [33.5-41.0] g/L; *p* = 0055).

It is worth noting that patients with diabetes presented abnormal levels of multiple indicators of heart function and inflammation. Inflammatory biomarkers, including high-sensitivity CRP (median [IQR], 34.6 [6.68-81.73] vs. 18.0 [2.15-55.0] mg/L; *p* = 0.0045) and globulin (median [IQR], 27.9 [25.8-32.5] vs. 26.4 [23.7-29.7] g/L; *p* < 0.0001) were significantly higher in patients with diabetes. Patients with diabetes also had significantly higher levels of cardiac injury biomarkers, including troponin T (TnT) (median [IQR], 0.013[0.009-0.032] vs. 0.008 [0.006-0.013] ng/mL; *p* < 0.0001), creatine kinase-myocardial band (CK-MB) test (median [IQR], 1.73 [0.97-3.51] vs. 1.19 [0.76-2.22] ng/mL; *p* = 0.0050), myoglobin (median [IQR], 49.6 [27.2-146.5] vs. 42.5 [26.2-76.1] ng/mL; *p* = 0.0121), and N-terminal pro-brain natriuretic peptide (NT-proBNP) (median [IQR], 271.9 [99.8-618.0] vs. 134.9 [48.6-422.9] pg/mL; *p* = 0.0037).

**Figure 2:**
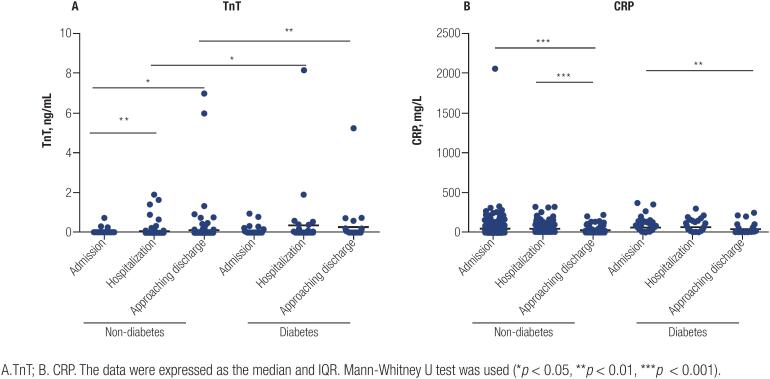
Dynamic change of TnT, and CRP during hospitalization. A. TnT; B. CRP. The data were expressed as the median and IQR. Mann-Whitney U test was used (*p < 0.05, **p < 0.01, ***p < 0.001).

We further analyzed the dynamic changes in TnT and CRP levels during hospitalization among these patient cohorts (Figure 2). As shown in Figure 2A, the TnT level of patients with diabetes increased significantly during the course of hospitalization compared to that in patients without diabetes (median [IQR], 0.013 [0.007-0.044] vs. 0.010 [0.006-0.019] ng/mL, *p* = 0.0167 at hospitalization and 0.030 [0.008-0.111] vs. 0.010 [0.005-0.0203] ng/mL, *p* = 0.0040 approaching discharge). Both groups of patients exhibited high CRP levels during the course of hospitalization (median [IQR], 19.1 [4.4-104.5] vs. 11.6 [2.6-49.2] mg/L, *p* = 0.1042 at hospitalization). The CRP levels of patients with diabetes were controlled close to the normal range (median [IQR], 5.7 [1.35-30.6] mg/L), with no significant difference compared to patients without diabetes (median [IQR], 3.5 [1.4-18.6] mg/L) when approaching discharge (Figure 2B).

### Treatments, complications, and clinical outcomes

The median time from symptom onset to admission was 10 (IQR, 7-15) days in patients with diabetes, similar to that in patients without diabetes (*p* = 0.7271; Table 1). There was also no significant difference in hospitalization time between the two groups. During hospitalization, patients with diabetes more frequently developed complications, including ARDS (20 [23.5%] vs. 41 [8.6%]; *p* = 0.0002), acute heart failure (10 [11.8%] vs. 15 [3.1%]; *p* = 0.0017), metabolic acidosis (8 [9.4%] vs. 11 [2.3%]; *p* = 0.0036), acute kidney injury (8 [9.4%] vs. 19 [4.0%]; *p* = 0.0477), and disseminated intravascular coagulation (DIC) (4 [4.7%] vs. 3 [0.6%]; *p* = 0.0118) compared to patients without diabetes (Table 1). There were no significant differences in the incidence of bacterial infection and acute liver injury between the two groups.

A total of 355 patients (62.9%) underwent respiratory support, with 305 (54.1%), 35 (6.2%), and 15 (2.7%) receiving nasal cannula, non-invasive ventilation, and invasive mechanical ventilation, respectively. Most patients received antiviral (351 [62.2%]) and antibacterial (407 [72.1%]) therapies during hospitalization. Chinese medicine, glucocorticoids, and immunoglobulins were administered to 343 (60.8%), 193 (34.2%), and 90 (16.0%) patients, respectively. Overall, the rates of these treatments did not differ significantly between patients with and without diabetes.

**Figure 3:**
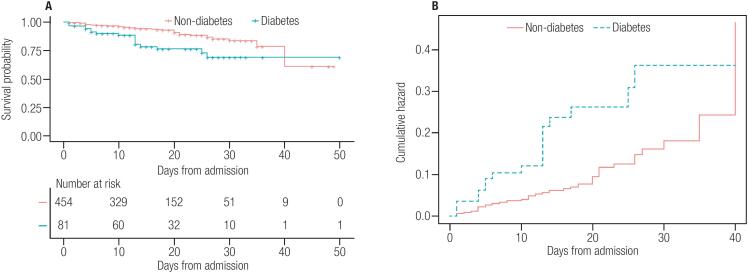
Kaplan-Meier plots of survival probability in hospitalized patients with COVID-19. **A.** Kaplan-Meier survival curves for mortality during the time from admission. Mortality was significantly higher in patients with diabetes. **B.** The hazard Function during the time from admission to 40-day follow up.

The relationship between diabetes and death was the focus of the present study. We found that diabetes was associated with a nearly 3.0-fold and significantly enhanced risk of death with COVID-19 (odds ratio [OR]: 2.950 [95% confidence interval (CI): 1.593-5.463], *p* <0.001), 0.001), which corresponded to an E-value of 5.348. The observed OR of 2.950 could be explained away by an unmeasured confounder that was associated with both diabetes and death by an odds ratio of 5.3-fold each, but weaker confounding could not do so. We excluded 29 patients (four with diabetes and 25 without) transferred to the superior hospital and selected confounders based on their associations with the outcomes of patients or a change in the effect estimate of at least 10% (Table 3). Multivariate logistic regression analysis indicated that the association between diabetes and outcome of COVID-19 did not change markedly after adjusting for hypertension, liver disease, and kidney disease (OR: 2.195 [95%CI: 1.048-4.599], *p* = 0.037, which produces E-value = 3.815 for the estimate. After adjusting for selected controlled confounders, the observed odds ratio of 2.195 could be explained away by an unmeasured confounder that was associated with both diabetes and death by an odds ratio of 3.8-fold each, above and beyond the measured confounders, but weaker confounding could not do so. Based on these 535 patients, we constructed a Kaplan-Meier survival curve. The results showed shorter durations from admission to 40-day follow-up in patients with diabetes compared to those without diabetes after giving a landmark of 40 (Figure 3A, mean = 31.621, SE = 1.704; mean = 36.008, SE = 0.633; *p* = 0.0012). The corresponding hazard function shown in Figure 3B indicates a higher probability of death in patients with diabetes than in patients without diabetes.

**Table 3:** Associations of covariates with the outcome of patients with COVID-19 (N = 535)^1^

		Status (0: survival; 1: dead)
Age, yr		0.099* (0.070-0.127)
Hypertension		
	Yes	0.878* (0.328-1.430)
	No	Reference
liver disease		
	Yes	1.343* (0.467-2.220)
	No	Reference
Kidney disease		
	Yes	1.924* (1.060-2.788)
	No	Reference

1 Values are regression coefficients (95% Confidence Interval) from univariate regression models and reflect differences in patients’ outcomes per unit change of each covariate and for different categories of each covariate as compared to the reference group.

* *P* value < 0.05.

### Characteristics of COVID-19 patients with diabetes with in-hospital death

As diabetes is an independent risk factor related to in-hospital death or poor prognosis in patients with COVID-19, we further analyzed the clinical characteristics of survivors and non-survivors of COVID-19. Among 85 COVID-19 patients with diabetes, those with older age and men were more likely to die. Compared to non-survivors with diabetes, survivors had longer hospitalization times (10 [4-13] vs. 19.5 [12-26] days, *p* < 0.0001). More non-survivors reported comorbidities related to cardiovascular disease (9, 52.9%) and malignancy (4, 23.5%). Non-survivors received fewer Chinese medicine (6 [35.3%] vs. 48 [70.6%], *p* = 0.0107), and more glucocorticoid treatment (12 [70.5%] vs. 22 [32.3%], *p* = 0.0057). Moreover, more non-survivors received mechanical ventilation. All these baseline characteristics interplayed the rapid progress of the disease in the COVID-19 patients with diabetes with in-hospital death (Table 4).

**Table 4:** The clinical characteristics and laboratory values of survivors and non-survivors in COVID-19 patients with diabetes

Characteristic		Survivors (n = 68)	Non-survivors (n = 17)	*P* value
Age-median (range)		62.5 (34-89)	73 (58-91)	0.0002
Male		36 (52.9)	13 (76.5)	0.1026
	Hospitalization, median (IQR), d	19.5 (12-26)	10 (4-13)	<0.0001
Comorbidity – No. (%)				
	Cardiovascular disease	16 (23.5)	9 (52.9)	0.034
	Cerebrovascular disease	1 (1.5)	2 (22.8)	0.1005
	Hypertension	45 (66.2)	13 (76.5)	0.5635
	Malignancy	1 (1.5)	4 (23.5)	0.0051
	Kidney disease	2 (2.9)	3 (17.6)	0.0523
Medical control for diabetes				
	No medication	28 (41.2)	8 (47.1)	0.7852
	Oral medication	37 (54.4)	3 (17.6)	0.0073
Insulin		21 (30.6)	8 (47.1)	0.2563
	Clinical treatment			
	Antiviral treatment	43 (63.2)	10 (58.8)	0.7837
	Antibiotics	49 (72.1)	13 (76.5)	1.0000
	Chinese Medicine	48 (70.6)	6 (35.3)	0.0107
	Glucocorticoid	22 (32.3)	12 (70.5)	0.0057
	Immune globulin	13 (19.1)	7 (41.1)	0.1056
Respiratory support				
	Nasal cannula	42 (61.8)	6 (35.3)	0.0005
	Non-invasive ventilation	3 (4.4)	7 (41.2)	
	Invasive ventilation	1 (1.5)	4 (23.5)	
Laboratory findings on admission				
	FBS (fasting blood glucose), mmol/L (normal range 3.9-5.8)	7.98 (6.08-12.62)	9.45 (6.53-14.20)	0.4692
	Arterial blood sugar, mmol/L (normal range 3.9-7.8)	9.30 (6.20-13.40)	10.65 (6.65-14.90)	0.3751
	White blood cell count, ×10^9^/L (normal range 3.5-9.5)	5.68 (4.37-7.54)	6.12 (3.60-7.99)	0.8372
	Neutrophil count, ×10^9^/L (normal range 1.6-6.3)	3.86 (2.99-5.97)	5.11 (3.05-7.18)	0.4207
	Lymphocyte count, ×10^9^/L (normal range 1.1-3.2)	0.99 (0.65-1.48)	0.53 (0.21-0.82)	0.0005
	Monocyte count, ×10^9^/L (normal range 0.1-0.6)	0.42 (0.28-0.52)	0.26 (0.15-0.34)	0.0066
	Platelet count, ×10^9^/L (normal range 125-350)	189.0 (135.5-251.0)	114.0 (82.0-152.5)	0.0003
Blood lipids and electrolytes				
	Total cholesterol, mmol/L (normal range 2.8-5.2)	3.48 (2.86-4.42)	3.31 (2.3-3.86)	0.2283
	Triglyceride, mmol/L (normal range 0.56-1.7)	1.01 (0.67-1.71)	1.19 (1.03-1.54)	0.1145
	HDL, mmol/L (normal range 0.9-2.1)	1.10 (0.85-1.25)	0.99 (0.81-1.24)	0.5957
	LDL, mmol/L (normal range 1-3.35)	2.07 (1.63-2.60)	1.60 (1.05-2.06)	0.0459
	sdLDL, mmol/L (normal range 95-538)	146.0 (101.3-244.8)	97.0 (65.0-151.0)	0.0127
	Potassium, mmol/L (normal range 3.5-5.3)	3.89 (3.57-4.33)	4.00 (3.11-4.89)	0.8854
	Calcium, mmol/L (normal range 2.11-2.52)	2.18 (2.02-2.31)	2.04 (1.98-2.18)	0.0748
	hsCRP, mg/L (normal range 0-3)	22.5 (4.3-64.2)	86.7 (46.3-123.7)	0.0002
	Procalcitonin, ng/mL (normal range 0-0.1)	0.059 (0.039-0.121)	0.227 (0.117-0.613)	0.016
	SAA, mg/L (normal range 0-10)	22.51 (8.71-62.91)	90.92 (79.51-180.8)	0.008
	TnT, ng/mL (normal range 0-0.014)	0.010 (0.008-0.018)	0.104 (0.027-0.275)	<0.0001
Creatine kinase-MB, ng/mL (normal range 0-6.22)		1.49 (0.78-2.87)	3.73 (1.48-16.22)	0.0095
	Myoglobulin, ng/mL (normal range 7.4-105.7)	39.8 (28.7-83.1)	145.9 (72.8-191.8)	0.0023
	NT-proBNP, pg/mL (normal range 0-222)	255.2 (93.9-399.5)	778.5 (343.6-9691)	0.0015
	PaO_2_, mmHg (normal range 70-107)	81.0 (65.5-108.5)	50.0 (39.5-61.3)	0.0004
	PaO_2_/FiO_2_, mmHg	350.0 (234.5-431.0)	131.0 (100.0-261.9)	0.0004
	PaCO_2_, mmHg (normal range 35-45)	39.0 (35.0-46.3)	31.0 (23.5-38.0)	0.0125
	PH (normal range 7.35-7.45)	7.41 (7.31-7.45)	7.41 (7.00-7.48)	0.9231
	BE, mmol/L (normal range -3-3)	1.40 (-0.9-3.13)	−3.65 (-5.28-0.18)	0.0001
	Alanine aminotransferase, IU/L (normal range 9-50)	22.0 (13.3-35.8)	27.0 (25.5-46.0)	0.0881
	Aspartate aminotransferase, IU/L (normal range 15-40)	26.0 (17.0-37.5)	35.0 (25.0-68.0)	0.0407
	Total protein, g/L (normal range 65-85)	63.6 (59.6-69.9)	63.8 (57.5-65.1)	0.2261
	Albumin, g/L (normal range 40-55)	36.3 (30.9-41.2)	32.5 (30.0-35.0)	0.0246
	Globulin, g/L (normal range 20-40)	27.6 (25.9-32.2)	29.0 (24.4-35.3)	0.5273
	Total bilirubin, μmol/L (normal range 2-23)	9.2 (7.2-12.4)	9.5 (7.5-19.88)	0.4404
	Direct bilirubin, μmol/L (normal range 0-8)	3.45 (2.2-5.33)	4.7 (3.2-8.8)	0.0512
	Creatinine, μmol/L (normal range 57-97)	66.0 (54.0-79.0)	68.0 (50.0-112.0)	0.8287
	Urea nitrogen, μmol/L (normal range 3.1-8)	4.71 (3.58-6.16)	6.95 (3.72-12.28)	0.0256

HDL: high-density lipoprotein; sdLDL: small dense low-density lipoprotein; LDL: low-density lipoprotein; CRP: C-reactive protein; SAA: serum amyloid A; TnT: troponin T; CK-MB: creatine kinase-MB; NT-proBNP: N-terminal pro-brain natriuretic peptide.

Compared to survivors, non-survivors presented lower lymphocyte (median [IQR], 0.53 [0.21-0.82] vs. 0.99 [0.65-1.48] x10^9^/L; *p* = 0.0005) and monocyte (median [IQR], 0.26 [0.15-0.34] vs. 0.42 [0.28-0.52] x10^9^/L; *p* = 0.0005) counts and higher levels of hsCRP (median [IQR], 86.7 [46.3-123.7] vs. 22.5 [4.3-64.2] mg/L; *p* = 0.0002), procalcitonin (median [IQR], 0.227 [0.117-0.613] vs. 0.059 [0.039-0.121] ng/mL; *p* = 0.016), and SAA (median [IQR], 90.92 [79.51-180.8] vs. 22.5 1 [8.71-62.91] mg/L; *p* = 0.008), which indicated that non-surviving patients with diabetes tended to present stronger inflammatory responses. Moreover, non-survivors with diabetes had lower PaO_2_ (median [IQR], 50.0 [39.5-61.3] vs. 81.0 [65.5-108.5] mmHg; *p* = 0.0004) and PaO_2_/FiO_2_ (median [IQR], 131.0 [100.0-261.9] vs. 350.0 [234.5-431.0] mmHg; *p* = 0.0004), which indicated that non-survivors developed more severe respiratory dysfunction. In addition, non-survivors with diabetes had multiple abnormal laboratory values related to cardiac, hepatic, and renal impairment, including higher levels of TnT, CK-MB, myoglobulin, NT-proBNP, aspartate aminotransferase, and urea nitrogen and lower levels of albumin (Table 5).

**Table 5:** The laboratory values of survivors and non-survivors in COVID-19 patients with diabetes

Characteristic		Median (IQR)		
		Survivors (n = 68)	Non-survivors (n = 17)	*P* value
FBS (fasting blood glucose), mmol/L (normal range 3.9-5.8)		7.98 (6.08-12.62)	9.45 (6.53-14.20)	0.4692
Arterial blood sugar, mmol/L (normal range 3.9-7.8)		9.30 (6.20-13.40)	10.65 (6.65-14.90)	0.3751
Blood cell count				
	White blood cell count, ×10^9^/L (normal range 3.5-9.5)	5.68 (4.37-7.54)	6.12 (3.60-7.99)	0.8372
	Neutrophil count, ×10^9^/L (normal range 1.6-6.3)	3.86 (2.99-5.97)	5.11 (3.05-7.18)	0.4207
	Lymphocyte count, ×10^9^/L (normal range 1.1-3.2)	0.99 (0.65-1.48)	0.53 (0.21-0.82)	0.0005
	Monocyte count, ×10^9^/L (normal range 0.1-0.6)	0.42 (0.28-0.52)	0.26 (0.15-0.34)	0.0066
	Platelet count, ×10^9^/L (normal range 125-350)	189.0 (135.5-251.0)	114.0 (82.0-152.5)	0.0003
Blood lipids and electrolytes				
	Total cholesterol, mmol/L (normal range 2.8-5.2)	3.48 (2.86-4.42)	3.31 (2.3-3.86)	0.2283
	Triglyceride, mmol/L (normal range 0.56-1.7)	1.01 (0.67-1.71)	1.19 (1.03-1.54)	0.1145
	HDL, mmol/L (normal range 0.9-2.1)	1.10 (0.85-1.25)	0.99 (0.81-1.24)	0.5957
	LDL, mmol/L (normal range 1-3.35)	2.07 (1.63-2.60)	1.60 (1.05-2.06)	0.0459
	sdLDL, mmol/L (normal range 95-538)	146.0 (101.3-244.8)	97.0 (65.0-151.0)	0.0127
Serum				
	Potassium, mmol/L (normal range 3.5-5.3)	3.89 (3.57-4.33)	4.00 (3.11-4.89)	0.8854
	Calcium, mmol/L (normal range 2.11-2.52)	2.18 (2.02-2.31)	2.04 (1.98-2.18)	0.0748
	Inflammatory biomarkers			
	hsCRP, mg/L (normal range 0-3)	22.5 (4.3-64.2)	86.7 (46.3-123.7)	0.0002
	Procalcitonin, ng/mL (normal range 0-0.1)	0.059 (0.039-0.121)	0.227 (0.117-0.613)	0.016
	SAA, mg/L (normal range 0-10)	22.51 (8.71-62.91)	90.92 (79.51-180.8)	0.008
Cardiac biomarkers				
	TnT, ng/mL (normal range 0-0.014)	0.010 (0.008-0.018)	0.104 (0.027-0.275)	<0.0001
	Creatine kinase-MB, ng/mL (normal range 0-6.22)	1.49 (0.78-2.87)	3.73 (1.48-16.22)	0.0095
	Myoglobulin, ng/mL (normal range 7.4-105.7)	39.8 (28.7-83.1)	145.9 (72.8-191.8)	0.0023
	NT-proBNP, pg/mL (normal range 0-222)	255.2 (93.9-399.5)	778.5 (343.6-9691)	0.0015
Blood gas analysis				
	PaO_2_, mmHg (normal range 70-107)	81.0 (65.5-108.5)	50.0 (39.5-61.3)	0.0004
	PaO_2_/FiO_2_, mmHg	350.0 (234.5-431.0)	131.0 (100.0-261.9)	0.0004
	PaCO_2_, mmHg (normal range 35-45)	39.0 (35.0-46.3)	31.0 (23.5-38.0)	0.0125
	PH (normal range 7.35-7.45)	7.41 (7.31-7.45)	7.41 (7.00-7.48)	0.9231
	BE, mmol/L (normal range -3-3)	1.40 (-0.9-3.13)	−3.65 (-5.28-0.18)	0.0001
Liver and renal function				
	Alanine aminotransferase, IU/L (normal range 9-50)	22.0 (13.3-35.8)	27.0 (25.5-46.0)	0.0881
	Aspartate aminotransferase, IU/L (normal range 15-40)	26.0 (17.0-37.5)	35.0 (25.0-68.0)	0.0407
	Total protein, g/L (normal range 65-85)	63.6 (59.6-69.9)	63.8 (57.5-65.1)	0.2261
	Albumin, g/L (normal range 40-55)	36.3 (30.9-41.2)	32.5 (30.0-35.0)	0.0246
	Globulin, g/L (normal range 20-40)	27.6 (25.9-32.2)	29.0 (24.4-35.3)	0.5273
	Total bilirubin, μmol/L (normal range 2-23)	9.2 (7.2-12.4)	9.5 (7.5-19.88)	0.4404
	Direct bilirubin, μmol/L (normal range 0-8)	3.45 (2.2-5.33)	4.7 (3.2-8.8)	0.0512
	Creatinine, μmol/L (normal range 57-97)	66.0 (54.0-79.0)	68.0 (50.0-112.0)	0.8287
	Urea nitrogen, μmol/L (normal range 3.1-8)	4.71 (3.58-6.16)	6.95 (3.72-12.28)	0.0256

HDL: high-density lipoprotein; sdLDL: small dense low-density lipoprotein; LDL: low-density lipoprotein; CRP: C-reactive protein; SAA: serum amyloid A; TnT: troponin T; CK-MB: creatine kinase-MB; NT-proBNP: N-terminal pro-brain natriuretic peptide.

## DISCUSSION

According to World Health Organization statistics, the number of globally confirmed cases reached 13,150,645 with 574,464 deaths in 216 countries by July 15, 2020 (15). Early studies indicated that diabetes was one of the most prevalent comorbidities in patients with COVID-19 (3,16). The present cohort study provided detailed clinical characteristics and risk factors associated with clinical outcomes in patients with COVID-19 with and without diabetes admitted to the No.7 Hospital of Wuhan between January 20 and March 15, 2020. The overall case fatality rate in mainland China was 5.4% (4,649 deaths out of 85,677 confirmed cases as of July 15, 2020) (15). In our study, the prevalence of diabetes in COVID-19 patients was 15.1%, consistent with previous reports of proportions of COVID-19 patients with diabetes ranging from 2.7% to 24.9% (17–19). The in-hospital mortality rate in patients with diabetes was markedly higher than that in patients without diabetes (20.0% vs. 7.9%, *p* = 0.0022), in line with previous findings. Our data demonstrated that diabetes can be considered a risk factor for death in patients with COVID-19 in Wuhan. Previous studies on the SARS pandemic indicated that diabetes, as well as fasting blood glucose (FBG) level, were associated with severe or lethal SARS-CoV infections (20). Diabetes was also reported to be a significant risk factor for both mortality and morbidity due to MERS-CoV (21,22). The presence of diabetes was also associated with worse clinical outcomes in patients infected with the H1N1 influenza A virus (17, 23). Combined with our data, the cumulative findings confirmed that patients with diabetes are more susceptible to certain infectious diseases.

In this retrospective cohort study, we demonstrated that diabetes was associated with a worse COVID-19 prognosis compared to patients without diabetes with COVID-19. In our study, COVID-19 patients with diabetes were more likely to have comorbidities of hypertension and cardiovascular disease. It is worth noting that diabetic patients presented with significantly higher neutrophil counts and lower lymphocyte counts, indicating that these COVID-19 patients with diabetes exhibited more severe lymphopenia (24). In addition, diabetic patients with COVID-19 presented higher hsCRP levels. These observations suggested that patients with diabetes were prone to more serious infections due to immune system imbalance, which is in with previous findings that patients with COVID-19 had higher levels of cytokines, including interleukin (IL)-2, IL-8, and tumor necrosis factor (TNF)-α (25).

Moreover, diabetic patients with COVID-19 also presented abnormal levels of multiple laboratory findings at hospital admission, including those related to heart (TnT, CK-MB, myoglobulin, and NT-proBNP), liver (albumin), kidney (urea nitrogen), and lung function (PaO_2_, PaO_2_/FiO_2_), indicating that SAR-CoV-2 infection may be related to progressive systemic injury in patients with diabetes. Corresponding to these findings, diabetic patients with COVID-19 were more likely to develop more complications, including ARDS, acute heart failure, metabolic acidosis, acute kidney injury, and DIC. Angiotensin-converting enzyme-2 (ACE-2) has been reported as a receptor for both SARS-CoV-2 and SARS-CoV (1). As an enzyme of the renin-angiotensin system (RAS), ACE2 is widely expressed in human tissues, including the lung, kidney, heart, digestive tract, blood vessels, testis, immune cells, and pancreas (26). Therefore, it is unsurprising that COVID-19 patients experience multiple extrapulmonary manifestations and possible complications. Yang and cols. demonstrated that the binding of SARS-CoV to ACE2 in pancreatic islet cells can lead to cell damage and acute diabetes (25). We assume that a similar mechanism related to pancreatic damage may also exist in COVID-19 infection, possibly contributing to hyperglycosemia, worse complications, and mortality, which requires further evidence.

In our study, 30.9% of the non-surviving COVID-19 patients had underlying diabetes. Among diabetic patients with COVID-19, there were more male non-survivors. Additionally, non-surviving COVID-19 patients with diabetes exhibited abnormal levels of biomarkers associated with severe forms of lymphopenia; inflammatory response; and cardiac, renal, hepatic, and respiratory system injury, consistent with previous reports (8,9). Combined with dynamic changes during hospitalization, surveillance of the levels of these biomarkers, especially cardiac indicators, may be helpful in the treatment of patients with COVID-19 patients with diabetes.

However, this study has several limitations. First, patients with uncomplicated illness were assigned to Fangcang shelter hospitals as an important component of the national response to the COVID-19 pandemic, which may have resulted in increased enrollment of patients with severe COVID-19. Second, the follow-up medical data were incomplete, as some cases were transferred. Third, this retrospective study relied on data collected from electronic medical records, from which some information was unavoidably missing.

The results of the present study suggested that diabetes was significantly associated with disease severity and fatal outcomes of COVID-19. Patients with COVID-19 patients with diabetes experienced severe multiple-organ manifestations and complications, especially myocardial and kidney injury, indicating the potential need for more intensive treatment and surveillance in these patients. Long-term observation and prospective study design are needed to assess the effectiveness of treatments specific for COVID-19 patients with diabetes.
